# Analysis and prediction of older adult sports participation in South Korea using artificial neural networks and logistic regression models

**DOI:** 10.1186/s12877-023-04375-2

**Published:** 2023-10-19

**Authors:** Hyun Byun, Sangwan Jeon, Eun Surk Yi

**Affiliations:** Department of Exercise Rehabilitation, Gachon University, 191 Hambakmoe-ro, Yeonsu-gu, Incheon, 21936 Republic of Korea

**Keywords:** Neural networks model, Logistic regression model, Older adult participants, Medical costs

## Abstract

**Background:**

Korea’s aging population and the lack of older adult participation in sports are increasing medical expenses.

**Aims:**

This study aimed to segment older adult sports participants based on their demographic characteristics and exercise practice behavior and applied artificial neural network and logistic regression models to these segments to best predict the effect of medical cost reduction. It presents strategies for older adult sports participation.

**Methods:**

A sample comprising data on 1,770 older adults aged 50 years and above was drawn from the 2019 National Sports Survey. The data were analyzed through frequency analysis, hierarchical and K-means clustering, artificial neural network, logistic regression, cross-tabulation analyses, and one-way ANOVA using SPSS 23 and Modeler 14.2.

**Results:**

The participants were divided into five clusters. The artificial neural network and logistic analysis models showed that the cluster comprising married women in their 60s who participated in active exercise had the highest possibility of reducing medical expenses.

**Discussion:**

Targeting women in their 60s who actively participate in sports, the government should expand the supply of local gymnasiums, community centers, and sports programs. If local gymnasiums and community centers run sports programs and appoint appropriate sports instructors, the most effective medical cost reduction effect can be obtained.

**Conclusions:**

This study contributes to the field by providing insights into the specific demographic segments to focus on for measures to reduce medical costs through sports participation.

##  Background

The development of science and technology and medical technology, as well as changes in the living environment, have enabled significant progress in addressing various diseases and improving people’s quality of life; this has dramatically increased the average life expectancy of humankind and created super-aged societies [1]. The National Statistical Office predicts that in 2070, the life expectancy of Koreans will be 89.5 years for men and 92.8 years for women, which is the highest among the OECD countries. Among major OECD countries, the aging of Korean society is progressing rather rapidly. In 2001, Korea entered the “aging society” category with its older adult population constituting 7.2% of its total population. In 2018, it remained in the “aging society” category with its older adult population constituting 14.4% of its total population. Korea is expected to enter the “super-aged society” category, as the ratio of the older adult population is expected to rise to 20.6% in 2025 [2]. The aging population problem in Asia has many side effects such as high morbidity, disability, and medical utilization rates [3, 4]. Many studies have reported that older adults’ sports participation has positive impacts in solving both their psychological and physical health problems [5–7]. Numerous studies have shown that physical activity among older adults effectively prevents various adult and cardiovascular diseases such as high blood pressure and obesity [8–10]. Gyasi et al. [11] stated that exercising helps alleviate loneliness among older adults by enhancing social connectivity. Older adults’ sports participation contributes positively toward their mental and physical health. An objective indicator that can measure the effectiveness of physical activity and older adults’ sports participation is the effect of reducing medical expenses [12]. Furukawa [13] showed that physical activity reduces medical expenses, diabetes, and hypertension in every household. Lobelo et al. [14] found that the participation of older adults in physical activity in the US and the UK can reduce social costs, especially medical spending.

Given that the engagement of older individuals in sporting endeavors contributes to addressing diverse health issues, it becomes essential to categorize the attributes of the older adult population and anticipate the impact of their involvement in sports activities [15]. Among the several types of prediction theories, some use machine learning whereas others rely on statistics. Predictive techniques that use machine learning include methods that rely on artificial neural networks and genetic algorithms. Statistics-based predictive techniques can be divided into logistic regression and time series analyses. First, the artificial neural network model, a representative predictive technique for machine learning, is extensively used for the control and optimization of industries, production processes, prediction, and pattern recognition [16]. They are mathematical structures that build neuron systems to make new decisions, and classify and predict using previously resolved results [17, 18]. However, studies related to artificial neural networks have shown that they have the disadvantage of being used when accurate prediction is needed rather than when the explanatory power for each variable is required, as they only provide prediction results but do not show which variables have significant effects on dependent ones and which interaction effects resulted in the outcome [19]. Meanwhile, logistic regression is used in the description and analysis of data to explain the relationship between one dependent binary variable and one or more independent variables [20]. Logistic regression is built upon specific assumptions concerning the data, including aspects such as independent observations and limited correlation between variables. Deviations from these assumptions can impact the integrity of the analysis. Recognizing that each model comes with its own inherent pros and cons, it becomes essential to conduct model comparisons to develop a prediction model that offers increased accuracy [21].

While prior research has contrasted predictive models within the medical domain, encompassing areas including mortality prognosis, length of hospital stays, and health-related consequences, limited attention has been directed toward the application and juxtaposition of diverse predictive models within the realm of sports [15].

Therefore, comparing predictive power, it is possible to introduce a method suitable for predicting the highest effect in medical cost reduction among older adults [22]. This study analyzes the older adults’ sports participation group with a high prediction rate for medical cost reduction, a target variable, using artificial neural network and logistic regression analysis models, that is, a machine-learning simulation and a statistical method, respectively. Additionally, identifying the characteristics of older adult sports participants and understanding the patterns of their participation is a crucial component of research on older adults’ sports behavior [23]. Rather than simply predicting consumer characteristics using artificial neural networks, classifying and subdividing these groups is a better way to increase the accuracy ratio [19, 24]. It is possible to identify the characteristics of groups with a high predictability of target variables [25]. Greater predictability and better results can be obtained by presenting artificial neural networks as a complementary means of cluster analysis; the artificial neural network model is the most promising field for sports consumer behavior analysis [26]. Although various studies have measured the effect of exercise on the reduction of medical expenses among older adults [27–29], few studies have categorized older adults based on their characteristics and exercise. Older adults are often considered a group with homogeneous characteristics and desires. However, given that this population comprises diverse sub-groups based on their health and employment status, among other aspects [30], it is necessary to classify them based on their demographic characteristics and exercise behavior.

**Fig. 1:**
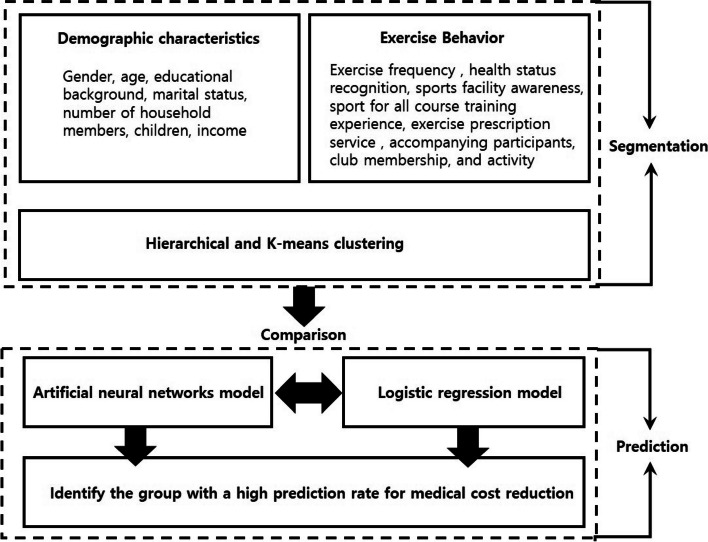
Research design and purpose

This study divides older adult sports participants into groups based on their demographic characteristics and exercise practice behavior. Second, artificial neural network and logistic regression models were applied to each group to identify the older adult sports participating group with the highest probability (classification accuracy rate) in the target variable (medical cost reduction). Third, the study aimed to analyze the characteristics of the group with the highest possibility of medical cost reduction. It also presents strategies to enhance older adults’ sports participation (Fig. 1).

## Methods

### Participants

 This study used data from the 2019 National Sports Survey conducted by the Ministry of Culture, Sports and Tourism. The sample size was 9,000, and Korean citizens aged 10 years were sampled. A random sampling of the number of households in each city and province in Korea was accomplished through a stratified multi-stage cluster sampling method. In this study, older adults aged 50 years and above were separated from the original data and identified as participants. Finally, 1,770 samples were used. Table 1 presents the demographic characteristics of the participants.

**Table 1:** Demographic characteristics of the study subjects

Variable		
Classification	N	%
Gender		
Male	831	46.9%
Female	939	53.1%
Age (years)		
50 to 59 years	430	24.3
60 to 69 years	663	37.5
70 years and above	677	38.2
Education level		
Elementary school	350	19.8
Junior high school	382	21.6
Senior high school	883	49.9
College/university	107	6.0
Postgraduate	48	2.7
Marital status		
Married	1563	88.3
Single	17	1.0
Widowed	154	8.7
Divorced	34	1.9
Others	2	.1
Number of people in the household		
1	183	10.3
2	1037	58.6
3	256	14.5
4	280	15.8
5	13	.7
Others	1	.1
Descendant		
None	166	9.4
1	339	19.2
2	802	45.3
3 and above	463	26.2
Income		
USD 800 to 1200	117	6.6
USD 1200 to 2000	213	12.0
USD 2000 to 2800	278	15.7
USD 2800 to 3600	534	30.2
USD 3600 to 4400	318	18.0
above USD 4400	310	17.5

### Variables

Various variables were selected from the 2019 National Sports Survey, such as gender, age, education, marital status, housing condition, number of descendants, and income levels. The main variables analyzed were gender, age, educational background, marital status, number of household members, children, income, exercise frequency, health status recognition, sports facility awareness, sport for all course training experience, exercise prescription service, accompanying participants, club membership, and activity.

and acts, as independent variables. The dependent variable was medical costs.

### Statistical analysis

The data were processed using SPSS 23 and Modeler 14.2. First, frequency analysis was conducted to identify the demographic characteristics of the older adults sports participants. Second, to divide them based on their demographic characteristics and exercise practice behavior, the variables were converted into standardized scores (Z score). Cluster analysis was performed by combining the first and second stages of hierarchical and K-means clustering, respectively. Third, to identify the group with the highest classification accuracy rate in medical cost reduction, the artificial neural network and logistic regression models were applied to each group. Finally, a Chi-square test and one-way analysis of variance (ANOVA) were conducted to identify the characteristics of the group with the highest classification accuracy rate in medical cost reduction.

## Results

### Cluster analysis

In previous studies focusing on cluster analysis, rather than selecting one method and deriving a result, a method of estimating the appropriate number of clusters by a hierarchical method and finally determining the number of clusters using a non-hierarchical method has been proposed [19, 31]. Therefore, the demographic characteristics and exercise practice behavior of older adult sports participants were selected as reference variables for the clusters. Hierarchical methods were deployed. Older adult sports participants were divided using non-hierarchical methods. It is difficult to apply a non-hierarchical method if the initial number of clusters is not known. Thus, hierarchical clusters were first executed to find the number of clusters [32]. A cluster analysis was conducted after converting the demographic (gender, age, educational background, marital status, number of household members, children, income) and sports practice (exercise frequency, health status recognition, sports facility awareness, sport for all course training experience, exercise prescription service, accompanying participants, club membership, and activity) variables to the standard score (Z score). First, for the hierarchical cluster analysis, the distance and average among the clusters were considered by analyzing the dendrogram. It was considered appropriate to determine the number of clusters within the range of 4−6.

 Next, K-means cluster analysis, a non-hierarchical method, was conducted on the range identified. As the K-means cluster analysis method is relatively easy for researchers to process large-scale data by designating reference variables and the number of clusters in advance [33–35], in this study, clusters were designated as 4, 5, and 6 based on the results of hierarchical cluster analysis. When four clusters were designated, the classification of clusters in recognition of sports facilities was insignificant (F = 2.274, p > .05). Thus, four clusters were not appropriate. When five clusters were designated, it was significant for all items, but the number of classified cases by cluster (cluster 1:172, cluster 2:138, cluster 3:161, cluster 4:709, cluster 5:590) differed. Accordingly, the number of clusters was designated as six; when this was analyzed, the distance between centers for each cluster was found to be more stable when five clusters were designated, and the final five clusters were determined (Table 2).

**Table 2:** Results of cluster analysis

Variable for segmentation	Type of elderly participants					Mean square	Mean error	F	p
	Cluster 1	Cluster 2	Cluster 3	Cluster 4	Cluster 5				
Gender	.340	.199	-.249	-.022	.035	8.765	.980	8.944	.000***
Age	.581	.275	-.006	-.356	1.004	156.393	.549	284.831	.000***
Education level	-.897	-.029	-.102	.209	-.774	98.384	.570	172.662	.000***
Marital status	2.946	.020	-.298	-.298	-.333	408.643	.160	2550.867	.000***
Number of people in the household	-1.459	-.280	.178	-.006	-.686	101.627	.502	202.369	.000***
Descendant	.072	.028	.149	-.496	.634	103.626	1.000	103.615	.000***
Income	-.752	.340	.540	.679	-.541	161.112	.634	254.216	.000***
Exercise frequency	.443	-.891	.002	-.197	.405	66.696	.821	81.193	.000***
Health status recognition	-.442	.101	.068	.375	-.322	49.263	.860	57.305	.000***
Sports facility recognition	-.017	-.282	-.311	-.238	-.118	3.345	.492	6.803	.000***
Sport for All course experience	.151	-1.582	.049	.086	.369	107.723	.672	160.253	.000***
Exercise prescription service	.146	-2.914	.310	.310	.286	326.427	.159	2047.741	.000***
Accompanying participants	-.129	.318	2.117	-.361	-.309	221.224	.526	420.495	.000***
Club membership and activities	.134	-1.107	-.660	.231	.221	76.880	.599	128.353	.000***
Number of cases by cluster	172	138	161	709	590	df=1765			

###  Artificial neural network model

 The application of the artificial neural network model proceeded as follows. First, the algorithm applied an equation for prediction. Second, parameter estimation was organized as a ratio of 70% training set and 30% test set. Third, the training method used sigmoid functions (activation functions characterized by collecting signal strengths from multiple neurons and converting them into numbers close to 1 as the signal strength becomes greater than 0, and vice versa [36]) that are commonly used in non-linear functions and artificial neural networks; the weights were designated as 0.9 to limit the demand for infinitely large weight values [37]. Fourth, the learning rate eta played a role in adjusting the weight modified in the process of finding the target variable by finding the direction to adapt to, and the artificial neural network model repeatedly, and this study was conducted by fixing it to the most commonly used eta value of 0.3 [38]. Fifth, the number of neurons in the hidden layer determined from the results were compared by applying the number of nodes in the hidden layer in various ways, such as 1, 2, 3, 4, 8, 16, and 32. In general, the rules for determining the number of neurons are as follows. First, “the number of hidden layer neurons is 2/3 of the size of the input layer” [39]. Second, “the number of neurons in the hidden layer must be less than twice the number of neurons in the input layer” [19]. Third, “the size of the hidden layer neuron is between the input layer size and the output layer size” [40]. Given that the number of input layers was 14 and the number of output layers was two, the most suitable number of hidden layers was identified as three. The study was conducted by designating all clusters as the final three hidden layers. These steps were applied to analyze the artificial neural network model for each cluster. Clusters 1 (60.45%), 2 (79.1%), 3 (66.8%), 4 (68.3%), and 5 (61.3%) had the highest possibilities of medical cost reduction (Table 3).

**Table 3:** Predictive probability (classification accuracy) analysis of medical cost reduction by cluster through an artificial neural network model

Classification		Cluster 1	Cluster 2	Cluster 3	Cluster 4	Cluster 5
Number of hidden layer fixed at 3		Medical cost reduction	Medical cost reduction	Medical cost reduction	Medical cost reduction	Medical cost reduction
Training	low possibility	67.7%	27.3%	70.5%	27.5%	28.3%
	high possibility	52.5%	98.7%	54.5%	89.2%	86.4%
	total	60.5%	82.5%	63.8%	66.7%	61.3%
Test	low possibility	78.3%	27.3%	81.3%	30.4%	31.6%
	high possibility	44.0%	96.3%	52.4%	93.8%	81.4%
	total	60.4%	75.7%	69.8%	69.9%	61.4%
Average classification accuracy rate		60.45%	79.1%	66.8%	68.3%	61.3%

###  Application of logistic regression analysis

Logistic regression analysis was performed along with the artificial neural network model to analyze the classification accuracy rate for medical cost reduction in each cluster. As the medical cost reduction effect (high group = 1, low group = 2) was set as a binary variable, it followed a binary distribution rather than a normal one as in general regression analysis. Similar to the artificial neural network model, logistic regression analysis does not directly predict whether the medical cost reduction effect is negative or positive but rather refers to the probability of how accurately it is predicted according to the low and high groups. The results of logistic regression analysis were evaluated for suitability through − 2 Log-likelihood verification (the lower, the better), Cox and Shell (the closer to 0, the better), standard error (the lower, the better), and Homer and Lemeshow (the less significant model) tests. The final classification accuracy rate was thus analyzed.

 Cluster-specific classification accuracy rates for medical cost reduction were as follows: 64.0% for cluster 1, 74.6% for cluster 2, 70.2% for cluster 3, 67.4% for cluster 4, and 59% for cluster 5. Both models identified cluster 2 as the group with the highest possibility of reducing medical expenses (Table 4).

**Table 4:** Predictive probability (classification accuracy) analysis of medical cost reduction by cluster through a logistic regression model

Classification		Cluster 1	Cluster 2	Cluster 3	Cluster 4	Cluster 5
Verification method	Standard	Medical cost reduction	Medical cost reduction	Medical cost reduction	Medical cost reduction	Medical cost reduction
-2 log-likelihood	the lower the better	215.57	149.913	171.744	889.063	777.771
Cox and Shell *R*^*2*^	close to zero is better	.124	.060	.249	.063	.043
Standard error	the lower the error, the more reliable	.153	.194	.160	.078	.083
Homer and Lemeshow test	p>.05	.702	.067	.220	.002	.045
Prediction of reduction in medical costs for each group (classification accuracy rate)	low possibility	69.3	8.3	72.6	25.1	25.3
	high possibility	58.3	98	66.7	92.4	83.9
	total	64.0	74.6	70.2	67.4	59.2

###  Understanding cluster characteristics

 To analyze the characteristics of cluster 2, which had the highest possibility of medical cost reduction, Chi-square test with other clusters and one-way ANOVA were performed. There were significant differences in the demographic and exercise practice variables (*p* < .001). It was found that 61.6% were women, 39.1% were in their 60s, and 54.3% were high school graduates. Further, 87.7% were married, 57.2% lived in a two-person household, and 57.2% had two children. Income was 35.5%, between 2.8 thousand and 3.6 thousand dollars. Further, 30.4% exercised more than thrice a week; 52.9% considered themselves healthy, and 97.8% were aware of the surrounding sports facilities. Additionally, 81.9% had experience teaching sports courses, and 91.3% had experience using exercise prescription services. As many as 36.2% participated in exercise alone, and 42.8% joined clubs (Table 5).

**Table 5:** Chi-square test results according to the demographic characteristics of each cluster

Classification		Cluster 1	Cluster 2	Cluster 3	Cluster 4	Cluster 5	x^2^	***P<.001
Gender	male	54 (31.4%)	53 (38.4%)	98 (60.9%)	351 (49.5%)	275 (46.6%)	35.165	.000***
	female	112 (68.6%)	85 (61.6%)	63 (39.1%)	358 (50.5%)	315 (53.4%)		
Age	50 to 59 years	25 (14.5%)	32 (23.2%)	54 (33.5%)	307 (43.3%)	12 (2.0%)	749.014	.000***
	60 to 69 years	54 (31.4%)	54 (39.1%)	67 (41.6%)	360 (50.8%)	128 (21.7%)		
	70 years and above	93 (54.1%)	52 (37.7%)	40 (24.8%)	42 (5.9%)	450 (76.3%)		
Education level	elementary school	89 (51.7%)	19 (13.8%)	18 (11.2%)	22 (3.1%)	202 (34.2%)	607.006	.000***
	junior high school	3620.9%)	24 (17.4%)	29 (18.0%)	77 (10.9%)	216 (36.6%)		
	senior high school	39 (22.7%)	75 (54.3%)	102 (80.3%)	499 (70.4%)	168 (28.5%)		
	college/university	4 (2.3%)	10 (7.2%)	9 (9.7%)	82 (11.6%)	2 (0.3%)		
	postgraduate	4 (2.3%)	10 (7.2%)	3 (4.4%)	29 (4.1%)	2 (0.3%)		
Marital status	married	0 (0%)	121 (87.7%)	159)98.8%)	694 (97.9%)	589 (99.8%)	860.013	.000***
	single	2 (1.2%)	2 (1.4%)	0 (0%)	12 (1.7%)	1 (0.2%)		
	widowed	135 (78.5%)	14 (10.1%%)	2 (1.2%)	3 (0.4%)	0 (0%)		
	divorced	33 (19.2%)	1 (2.9%)	0 (0%)	0 (0%)	0 (0%)		
	others	2 (0.2%)	0 (0%)	0 (0%)	0 (0%)	0 (0%)		
Number of people in the household	1	143 (83.1%)	9 (6.5%)	1 (0.6%)	11 (1.6%)	19 (3.2%)	1460.332	.000***
	2	17 (9.9%)	79 (57.2%)	71 (44.1%)	344 (48.5%)	526 (89.2%)		
	3	11 (6.4%)	28 (20.3%)	25 (15.5%)	155 (21.9%)	37 (6.3%)		
	4	1 (0.6%)	19 (13.8%)	60 (37.3%)	192 (27.1%)	8 (1.4%)		
	5	0 (0%)	2 (1.4%)	4 (2.5%)	7 (1.0%)	0 (0%)		
	others	0 (0%)	1 (0.7%)	0 (0%)	0 (0%)	0 (0%)		
Descendant	none	19 (11%)	8 (5.8%)	9 (5.6%)	112 (15.8%)	18 (3.1%)	433.255	.000***
	1	35 (20.3%)	30 (21.7%)	20 (12.4%)	203 (28.6%)	51 (8.6%)		
	2	60 (34.9%)	71 (51.4%)	98 (60.9%)	357 (50.4%)	216 (36.6%)		
	above 3	58 (33.7%)	29 (21%)	34 (21.1%)	37 (5.2%)	305 (51.7%)		
Income	USD 800 to 1200	55 (32%)	5 (3.6%)	6 (3.7%)	2 (0.3%)	49 (8.3%)	860.013	.000***
	USD 1200 to 20000	40 (23.3%)	8 (5.8%)	8 (5%)	15 (2.1%)	142 (24.1%)		
	USD 2000 to 2800	25 (14.5%)	15 (10.9%)	25 (15.5%)	43 (6.1%)	170 (28.8%)		
	USD 2800 to 3600	31 (18%)	49 (35.5%)	36 (22.4%)	222 (31.3%)	196 (33.2%)		
	USD 3600 to 4400	14 (8.1%)	34 (24.6%)	19 (11.8%)	226 (31.9%)	25 (4.2%)		
	above USD 4400	7 (4.1%)	27 (19.6%)	67 (41.6%)	201 (28.3%)	8 (1.4%)		
Exercise frequency	less than once a week	47 (27.3%)	18 (13%)	82 (50.9%)	181 (25.5%)	117 (19.8%)	89.225	.000***
	twice a week	31 (18%)	36 (26.1%)	15 (9.3%)	140 (19.7%)	116 (19.7%)		
	thrice a week	31 (18%)	42 (30.4%)	30 (18.6%)	160 (22.6%)	144 (24.4%)		
	four times a week	15 (8.7%)	6 (4.3%)	7 (4.3%)	56 (7.9%)	43 (7.3%)		
	more than 5 times a week	48 (27.9%)	36 (26.1%)	27 (16.7%)	172 (24.3%)	170 (28.8%)		
Health status recognition	not very healthy	5 (2.9%)	0 (0%)	1 (0.6%)	1 (0.1%)	3 (0.5%)	244.118	.000***
	not healthy	48 (27.9%)	16 (11.6%)	19 (11.8%)	31 (4.4%)	131 (22.2%)		
	normal	65 (37.8%)	43 (31.2%)	52 (32.3%)	208 (29.3%)	265 (44.9%)		
	healthy	44 (25.6%)	73 (52.9%)	81 (50.3%)	384 (54.2%)	164 (27.8%)		
	very healthy	10 (5.8%)	6 (4.3%)	8 (5.0%)	85 (12%)	27 (4.6%)		
Sports facility recognition	aware	154 (89.5%)	135 (97.8%)	159 (98.8%)	684 (96.5%)	547 (92.7%)	26.875	.000***
	unaware	18 (10.5%)	3 (2.2%)	2 (1.2%)	25 (3.5%)	43 (7.3%)		
Sport for all course experience	experienced	23 (13.4%)	113 (81.9%)	28 (17.4%)	113 (15.9%)	28 ( (4.7%)	471.566	.000***
	inexperienced	149 (86.6%)	25 (18.1%)	133 (82.6%)	596 (84.1%)	562 (95.3%)		
Exercise prescription service	experienced	8 (4.7%)	126 (91.3%)	0 (0%)	0 (0%)	4 (0.7%)	1456.213	.000***
	inexperienced	164 (95.3%)	12 (8.7%)	161 (100%)	709 (100%)	586 (99.3%)		
Accompanying participants	alone	85 (49.4%)	50 (36.2%)	0 (0%)	351 (49.5%)	290 (49.2%)	1208.565	.000***
	family	20 (11.6%)	10 (7.2%)	0 (0%)	159 (22.4%)	105 (17.8%)		
	friend	51 (29.7%)	43 (31.2%)	12 (7.5%)	189 (26.7%)	186 (31.5%)		
	colleague	1 (0.6%)	3 (2.2%)	9 (5.6%)	10 (1.4%)	2 (0.3%)		
	club	3 (1.7%)	15 (10.9%)	32 (19.9%)	0 (0%)	1 (0.2%)		
	local resident	12 (7%)	17 (12.3%)	107 (66.5%)	0 (0%)	6 (1%)		
	others	0 (0%)	0 (0%)	1 (0.6%)	0 (0%)	0 (0%)		
	unregistered	167 (97.1%)	78 (56.5%)	116 (72%)	688 (97%)	579 (98.1%)		
	no activities though registered	0 (0%)	1 (0.7%)	0 (0%)	11 (1.6%)	5 (0.8%)		

Next, one-way ANOVA was conducted to understand the differences in the demographic characteristics and exercise practice behavior in each cluster; significant differences were identified (*P* < .05). In cluster 2, the experience of sports courses, use of exercise prescription services, club membership, and activities were significantly higher than those of other clusters. Cluster 2 was called “A group of married women in their 60s who actively participated in sports,” following a comparison of the results and demographic characteristics as well as exercise behavior variables.

Cluster 1 was a group of women with low income who lived alone, named “A group of women in their 70s, living alone.” Cluster 3 participated in sports less than once a week; had high income; and were in their 60s, married, and men, named a “A group of married men in their 60s with insufficient exercise.” Cluster 4 was a group of married women in their 60s who exercised more than thrice a week, named “A group of married women in their 60s who exercised regularly.” Cluster 5 was a group of married women in their 70s who exercised more than thrice a week, named “A group of married women in their 70s who exercised regularly.”

## Discussion

Previous research [41, 42] on older adults’ healthcare shows that using market segmentation and artificial neural network models can specifically grasp the characteristics cluster. Launay et al. [43] predicted the long-term hospitalization of older adults using an artificial neural network because they obtained more accurate classifications for the target variable. To analyze consumers more specifically through several studies, classifying them and identifying groups with high prediction rates for specific variables among each group is the best way to predict behavior [24]. Therefore, this study classified older adult sports participants through K-means clustering and applied artificial neural network and logistic regression models to these clusters to predict their medical cost reduction rates with high accuracy and obtained the following results [18].

First, the classification results of each cluster were statistically significant. Each characteristic was well depicted. The artificial neural network model showed higher classification accuracy rates than the logistic regression model. These results are consistent with Lin et al. [20]. Zhao et al. [44] classified target customers in distribution industry marketing into three groups and compared and analyzed the classification accuracy rate using statistical methods, logistic regression, and artificial neural networks. They compared artificial neural networks and logistic regression analysis using explanatory variables such as shopping mall residence time, flow direction, shopping background, and revisit count data. Artificial neural network analysis obtained a 5.26% improvement in prediction results when compared to logistic regression, indicating that the classification accuracy rate of the artificial neural network model was the best and consistent with the results of this study. Hosseini et al. [45] divided patients into five groups to analyze the classification accuracy rate, using the patients’ recent lookups, the period for which they relied on the hospital for services, the number of visits they made, and the total fees they paid as variables. The artificial neural network analysis had excellent predictive power with 89.31%, indicating that the results were supported.

Liou et al. [46] utilized duration of drug dispensation, drug cost, consultation and treatment, diagnosis, dispensing service fees, medical expenditure, amount claimed, drug cost per day, and medical expenditure per day as variables to compare the classification accuracy rate for fraud prediction. Consequently, 96% accuracy rate of artificial neural networks and 92% accuracy rate of logistic regression analysis were found, indicating that the artificial neural network model showed better predictive power than logistic regression analysis. Studies have shown that the artificial neural network model is superior to existing statistical methods of predicting consumer behavior [47–49]. In the current study, more specific predictions and various analysis methods were applied to examine older adult sports participants (specifically the group with the highest medical cost reduction effect). The data can be used to establish welfare policies for older adults.

Second, the artificial neural network and logistic regression models were applied to all groups to analyze the cluster with the highest classification accuracy rate in medical cost reduction. The results showed that cluster 2 had the highest classification accuracy rate, and the Chi-square test and one-way ANOVA helped identify the characteristics of the cluster. It was named “A group of married women in their 60s who exercised actively.” These results were supported by Zhu et al.’s [50] study on women’s exercise perseverance and barriers to exercise; they found that women in their 60s and who are high school graduates had the highest averages of exercise endurance, emphasizing that older adult participants should be divided into optimal categories from a long-term perspective. Griffin et al. [51] stated that owing to the differences in classified characteristics by cluster, it is possible to identify the older adults with the most significant risk to their physical and psychological health according to the characteristics of each cluster. Therefore, this study can also implement an exercise participation strategy suitable for each cluster based on the evident characteristics of cluster 2. For example, cluster 2 (a group of married women in their 60s who exercised actively) exercised more than thrice a week, recognized themselves as healthy, participated in the Sport for All course and exercise prescription services, and had relatively active club activities. Accordingly, the government will be able to attract the participation of older adults in sports through local gymnasiums and community centers and meet their various needs by expanding the supply of sports programs. Based on the results, demand analysis for exercise programs among participants of a specific age should be conducted to provide appropriate programs. The Korean government fosters professional human resources through the “Sports for All Instructor Qualification System” and reflects the characteristics of each age group in its sports policy through a survey on its use. According to Sevick et al. [52], the number of visits to medical institutions among the participating older adults was 12% lower than that of the non-participating older adults. The government would reduce medical costs by providing sports programs and instructors with expertise to welfare centers and national sports centers that may interest older adults in their 60s who actively participate in sports.

Although cluster 1 showed a lower classification accuracy for medical cost reduction when compared to cluster 2 through the artificial neural network and logistic regression models, the characteristics of older adult sports participants can be analyzed through cluster analysis. In cluster 1 (a group of women in their 70s, living alone with low income), health status was recognized as normal and the existence of sports facilities was recognized by many; however, their participation in the Sports for All course, use of exercise prescription services, and club membership and engagement in club activities were low. According to Statistics Korea [2], 32.2% of groups aged 70 years and above did not use sports facilities when compared to other groups, possibly because this group had a lower income when compared to other groups. Thus, the fee for using sports facilities was borne. Currently, many local governments in Korea are continuously investigating the adequacy of public sports facility fees. Accordingly, sports facility fees for older adults are reduced or free of charge based on the investigation. Hence, to enhance the usage frequency of older adult facilities, it becomes imperative to establish distinct pricing structures for each age subgroup within the older adult demographic, rather than adopting a uniform flat fee. Cluster 3 (men in their 60s with insufficient exercise) had higher income than other groups owing to demographic characteristics; participated in sports facilities less than once a week; and showed a lower participation rate of Sport for All course experience, exercise prescription service, and club activities. According to Han et al. [53], men with higher incomes are at a greater susceptibility to adult-onset ailments due to inadequate physical activity and unhealthy dietary practices. For older adults and individuals aged above 60 years with higher income, the incidence of social isolation-induced loneliness surpasses that of other demographic segments [54]. Thus, the implementation of exercise initiatives encompassing social interactions becomes imperative. However, senior citizen centers, and welfare and sports centers do not run programs for older adults that would allow them to communicate and create networks among themselves. High-income groups comprising men in their 60s are highly likely to participate in sports in the future given that they have stable incomes; thus, sports programs that can increase social communication with local residents should be provided. Clusters 4 and 5 had common characteristics as they comprised married women who exercised regularly, who were in their 60 and 70 s, respectively. They exercised more than thrice a week, and most of them stated that their health status was normal. Their participation in exercise prescription services and Sports for All courses, enrollment in club membership, and engagement in activities were low. They exercised actively, but the utilization rate of sports facilities and government-supported programs was low because facilities and programs for older adults were not sufficiently established. Owing to a lack of policy, the limited facilities in operation were also deficient and were left unattended. Sports facilities for older adults in Korea are operated without any distinction from professional sports facilities, sports facilities, and sports facilities at work in terms of installation and operation. According to the Ministry of Culture, Sports and Tourism [55], there are 30,185 public sports facilities nationwide, but facilities for physical education for older adults are not separated. The Ministry of Culture, Sports and Tourism [55] reported that at least 1,742 gateball courts are used by older adults, and 147 ground and park golf courses are operated, accounting for only 6.25% of the total. The physical structures and health conditions of older adults differ from those of young people, so sports programs must be tailored to suit their needs and specialized facilities must be established. Sport England operates a separate Active Aging fund to address mental health, dementia, and loneliness among older adults. The Netherlands recommends physical fitness tests for older adults with a focus on More Exercise for Seniors (MBvO), and Australia operates an Active Over 50 well-aging program. In Korea, National Physical Education 100 is operated as a sports welfare service that measures and evaluates physical fitness status and provides exercise, counseling, and prescriptions at a state-designated public certification agency. This service provides customized exercise programs based on an individual’s physical strength so that they can participate comfortably. It issues a national certificate that incentivizes participation among older adults. However, as the results of this study show, while facilities and sports programs are well equipped, the older adults’ low exercise prescription service utilization rate and Sport for All course experience suggest that there is a need to strengthen promotional and marketing activities for older adults.

## Conclusion

This study divided older adult sports participants based on their demographic characteristics and exercise practice behavior. The artificial neural network and logistic regression models were applied to each group to identify the older adult exercise participant group with the highest possibility for medical cost reduction. The study sought to analyze the characteristics of the group with the highest target variable and present a strategy to enhance older adults’ sports participation. First, the older adult sports participants were classified into five clusters. Second, the artificial neural network model showed that cluster 2 had the highest possibility of medical cost reduction. Third, the logistic regression model also showed that cluster 2 had the highest possibility of medical cost reduction. Fourth, a comparison of the results for cluster 2 drawn from applying both models showed that the group of married women in their 60s actively participated in exercise. Therefore, to maintain and manage this group, if the government uses local gymnasiums and community centers as supply bases for sports programs and conducts various programs with appropriate Sports for All instructors, the group’s medical cost reduction effect will be high.

This study had the following limitations, which can serve as recommendations for future research. First, owing to the lack of prior research in the field and the use of artificial neural networks and logistic regression models, several variables were excluded from the study. Therefore, future research should include more variables related to older adult sports participants (variables inducing sports participation, such as motivation to participate), and more diverse and detailed characteristics should be analyzed. Second, as the study was conducted using data from the National Sports Survey in 2019, it did not explain older adults’ sports participation and the resulting effect of exercise in light of the COVID-19 pandemic. Therefore, it is somewhat unreasonable to generalize the findings to the current state of older adult sports participants. In follow-up research, more detailed and specific groups can be identified to assess if the effect of medical expenditure on older adult sports participants is predicted by reflecting the latest data after COVID-19 onset. Cluster analysis can reduce a wide range of data and divide sports participants based on common characteristics, thereby offering more detailed results by drawing upon big data from government units.

## Data Availability

This work was prepared by the Ministry of Culture, Sports and Tourism in 2020 and used the 2020 National Sports Survey, which was opened as the fourth type of public Nuri; the work can be downloaded free of charge from the Ministry of Culture, Sports and Tourism website: https://www.mcst.go.kr.
